# In Vitro Antioxidant and Cytotoxic Activities of 18 Plants from the Erkowit Region, Eastern Sudan

**DOI:** 10.1007/s13659-018-0155-0

**Published:** 2018-02-16

**Authors:** Manar Adam, Gihan O. M. Elhassan, Sakina Yagi, Fatma Sezer Senol, Ilkay Erdogan Orhan, Abdel Azim Ahmed, Thomas Efferth

**Affiliations:** 1 Department of Botany, Faculty of Science, University of Khartoum, Khartoum, Sudan; 2 Department of Pharmacognosy, Faculty of Pharmacy, Gazi University, 06330 Ankara, Turkey; 3 Department of Pharmaceutical Biology, Johannes Gutenberg University, 55128 Mainz, Germany

**Keywords:** Antioxidant activity, Cytotoxicity, Pharmacognosy, Plant extract

## Abstract

We investigated the antioxidant potential and cytotoxicity towards human CCRF-CEM leukemia cells of 57 extracts obtained from 18 plants collected in the Erkowit region, eastern Sudan. The antioxidant activity was determined by measuring the radical scavenging effects against 2,2-diphenyl-1-picrylhydrazyl (DPPH) and *N*,*N*-dimethyl-*p*-phenylendiamine (DMPD), metal-chelation capacity, ferric-reducing (FRAP) and phosphomolibdenum-reducing antioxidant power (PRAP) methods using ELISA microtiter assays. Total phenol and flavonoid amounts of the extracts were determined spectrophotometrically. Cytotoxicity towards CCRF-CEM cells was evaluated by the resazurin reduction assay. *Geranium favosum* followed by *Kalanchoe glaucescens, Malva parviflora, Aizoon canariense*, and *Coleus barbatus*, respectively, possessed the highest antioxidant activity among the studied plants. *Chrozophora oblongifolia* and *K. glaucescens* exerted considerable cytotoxicity against CCRF-CEM leukemia cells. These plants may serve as source for the further development of natural antioxidant and antitumor agents.

## Introduction

Many terrestrial plants have been subjected to chemical and pharmacological screening, in order to evaluate their potential as drugs in medicine. Natural products are important sources for new pharmaceutical compounds. The ethnomedicinal approach represents an important method for identifying biologically active plant-based natural products as well as a means of documenting and preserving local knowledge [1]. Albuquerque et al. [2] discussed the limitations of ethnopharmacology and ethnobotany for the selection of plants for phytochemical and pharmacological screening. However, many studies have highlighted the efficiency of random screening approaches of plants to identify plants with therapeutic potential. For instance, Khafagi and Dewedar [3] found that random screening led to the identification of a large percentage of species (13.9%) with strong antimicrobial activity in comparison to that obtained (8.3%) from ethno-directed approaches. The same observation was reported by Gyllenhaal et al. [4] on their antitumor screening of plants against human MCF-7 breast cancer cells.

Oxidants are ubiquitous in biological systems and can cause significant damage to membranes, proteins, and nucleic acids. Plants with antioxidant activity lower the risk for ROS-mediated chronic diseases, such as cancer, ulcer, diabetes and cardiovascular disease [5–10].

Currently, the prevalence of cancer is estimated at 12.7 million people in 2008 and is expected to rise to 21.4 million by 2030. Nearly two-thirds of all cancer diagnoses occur in developing countries [11, 12]. A major limitation in cancer treatment is the development of drug resistance. Plant extracts contain a great diversity of bioactive compounds with multiple targets and mechanisms of action. Therefore, they could decrease the probability of emergence of resistant tumor clones [13–15].

Sudan has a rich biodiversity and a large number of medicinal herbs. The flora of Erkowit, eastern Sudan, comprises species from the Sahel zone, the Afro-montane domain, and a few with Mediterranean affinities [16]. Lack of information regarding local medicinal plants in this region limits their use as important health and economic components.

## Results

The selected plants from the Erkowit region in eastern Sudan have been surveyed for their traditional use. Some but not all of these plants have documented traditional uses. However, they are frequently used by traditional healers of the region. The identities, parts used and medicinal uses of the investigated plants are shown in Table 1.

**Table 1:** Profile of the investigated plants from the Erkowit region, eastern Sudan

Scientific names	Family	Voucher no.	Hedendowan name^a^	Part used	Traditional use^b^	Ref.
*Adiantum incisum* Forssk	Pteridaceae	AD/E15	=	Whole plant	Cough fever	[52]
*Anaptychia ciliaris* (L.) Körb.	Physciaceae	AC/E15	Bakour	Whole plant	Incense	
*Aizoon canariense* L.	Aizoaceae	ACa/E15	Gadkaheeb	Whole plant	Tumors	[53]
*Capparis decidua* (Forsk) Edgew.	Capparidaceae	CD/E15	Sarroub	Twigs	Swelling, joint pains, head-ache	[54]
*Chrozophora oblongifolia* (Del.) Adr. Juss. ex Spreng.	Euphorbiaceae	CO/E15	Koreeb		Gonorrhea	[55]
*Coleus barbatus* (Andrews) Benth.	Lamiaceae	CB/E15	Khahab	Whole plant	Allergy	TH^b^
*Forsskaolea tenacissima* L.	Urticacea	FT/E15	Lusaig		No use	
*Geranium favosum* Hochst. ex A.Rich.	Geraniaceae	GF/E15	Tawtaw		No use	
*Kalanchoe glaucescens* Britten	Crassulaceae	KG/E15	Hrfifoit	Whole plant	No use	
*Lavandula stricta* Del.	Lamiaceae	LS/E15	Sadam		No use	
*Leucas nubica* Benth.	Lamiaceae	LN/E15	Mayoub	Whole plant	Jaundice	TH
*Malva parviflora* L.	Malvaceae	MP/E15	Humaad	Leaves	Wounds	TH
*Oxalis anthelmintica* A. Rich	Oxalidaceae	OA/E15	Taitarob		No use	
*Rumex vesicarius* L.	Polygonaceae	RV/E15	Kobsa		No use	
*Scrophularia arguta* Soland	Scrophulariaceae	SA/E15	Cashaitleam		No use	
*Senna alexandrina* Mill.	Leguminosae	SAl/E15	Amerkeet	Fruits	Diabetes	TH
				Fruits, leaves	Constipation	[56]
*Trianthema portulacastrum* L.	Aizoaceae	TP/E15	Rabaa	Whole plant	Ulcers	TH
*Umbilicus botryoides* Hochst. ex A. Rich.	Crassulaceae	UB/E15	Buscolai	Whole plant	Wounds	TH

^a^Major tribe that lives in Erkowit region of Sudan

^b^*TH* traditional healers

In the current study, 57 extracts (dichloromethane (DMC), ethyl acetate (EtOAc) and methanol (MeOH) extracts) of 18 different Sudanese plants were tested for their potential antioxidant activity using five methods, namely 2,2-diphenyl-1-picrylhydrazyl radical scavenging (DPPH), metal chelation, ferric-reducing antioxidant power (FRAP), *N*,*N*-dimethyl-*p*-phenylendiamine (DMPD) and phosphomolibdenum-reducing antioxidant power (PRAP). The results are presented in Table 2. The radical scavenging ability measured by DPPH assay showed that extracts exhibited extremely large variation in their capacity to inhibit DPPH from 3.76 ± 0.30 to 92.07 ± 0.00%. The highest radical scavenging effect was observed with the MeOH extracts of *G. favosum* (92.07 ± 0.00%) and *K. glaucescens* (72.95 ± 0.04%) respectively.

**Table 2:** Antioxidant activity of the investigated plants extracts from the Erkowit region, eastern Sudan

Plant names	Extract type	Radical scavenging activity (% ± S.D.)^a^		Metal-chelation capacity (% ± S.D.)	FRAP^b^	PRAP^b^	Total phenol amount (mg/g)^c^	Total flavonoid amount (mg/g)^d^
		DPPH	DMPD					
*Adiantum incisum*	DCM	7.43 ± 0.01	4.77 ± 0.32	-^e^	0.301 ± 0.01	0.265 ± 0.01	0.169 ± 0.01	0.759 ± 0.02
	EtOAc	5.58 ± 0.06	1.53 ± 0.35	–	0.328 ± 0.02	0.281 ± 0.01	0.195 ± 0.12	1.142 ± 0.02
	MeOH	7.43 ± 0.04	4.77 ± 0.32	–	0.301 ± 0.01	0.265 ± 0.01	0.306 ± 0.01	0.311 ± 0.03
*Anaptychia ciliaris*	DCM	8.80 ± 0.06	–	52.29 ± 0.01	0.552 ± 0.04	0.649 ± 0.03	0.509 ± 0.01	0.516 ± 0.02
	EtOAc	17.12 ± 0.01	–	62.99 ± 0.04	0.450 ± 0.01	0.284 ± 0.09	0.389 ± 0.02	1.032 ± 0.01
	MeOH	14.62 ± 0.02	31.52 ± 0.26	40.86 ± 0.03	0.292 ± 0.02	0.294 ± 0.06	0.176 ± 0.01	0.293 ± 0.00
*Aizoon canariense* fruit	DCM	6.26 ± 0.03	–	48.03 ± 0.04	0.300 ± 0.00	0.183 ± 0.00	0.137 ± 0.03	0.183 ± 0.01
	EtOAc	6.80 ± 0.03	–	25.34 ± 0.13	0.301 ± 0.01	0.165 ± 0.02	0.162 ± 0.12	0.406 ± 0.04
	MeOH	36.09 ± 0.41	45.63 ± 0.23	68.33 ± 0.43	0.236 ± 0.01	0.178 ± 0.00	0.132 ± 0.01	0.129 ± 0.01
*Aizoon canariense*	DCM	3.84 ± 0.29	–	7.66 ± 0.02	0.309 ± 0.07	0.417 ± 0.02	0.278 ± 0.01	0.106 ± 0.01
	EtOAc	6.66 ± 0.04	–	–	0.367 ± 0.01	0.357 ± 0.00	0.247 ± 0.02	1.138 ± 0.47
	MeOH	20.19 ± 0.01	29.32 ± 0.25	37.25 ± 0.00	0.321 ± 0.01	0.232 ± 0.01	0.249 ± 0.12	0.676 ± 0.01
*Capparis decidua*	DCM	9.01 ± 0.01	13.56 ± 0.28	56.42 ± 0.49	0.377 ± 0.02	0.181 ± 0.00	0.262 ± 0.08	0.526 ± 0.08
	EtOAc	9.88 ± 0.01	26.28 ± 0.23	15.07 ± 0.04	0.298 ± 0.00	0.120 ± 0.01	0.140 ± 0.01	0.515 ± 0.02
	MeOH	21.13 ± 0.06	45.44 ± 0.20	8.76 ± 0.02	0.410 ± 0.02	0.265 ± 0.01	0.293 ± 0.01	0.162 ± 0.021
*Chrozophora oblongifolia*	DCM	12.95 ± 0.03	–	14.75 ± 0.08	0.450 ± 0.12	0.211 ± 0.00	0.182 ± 0.02	1.053 ± 0.05
	EtOAc	11.06 ± 0.02	0.97 ± 0.37	10.11 ± 0.08	0.299 ± 0.02	0.296 ± 0.01	0.200 ± 0.02	0.119 ± 0.12
	MeOH	8.87 ± 0.00	–	5.01 ± 0.04	0.390 ± 0.00	0.245 ± 0.00	0.194 ± 0.01	0.390 ± 0.01
*Coleus barbatus*	DCM	28.98 ± 0.09	–	12.28 ± 0.08	0.668 ± 0.02	0.251 ± 0.01	0.519 ± 0.01	0.372 ± 0.01
	EtOAc	23.12 ± 0.06	19.74 ± 0.28	67.23 ± 0.06	0.469 ± 0.01	0.280 ± 0.00	0.332 ± 0.02	1.129 ± 0.47
	MeOH	56.00 ± 0.03 1	52.17 ± 0.18	5.37 ± 0.10	0.850 ± 0.01	0.205 ± 0.01	0.641 ± 0.02	0.511 ± 0.01
*Forsskaolea tenacissima*	DCM	5.39 ± 0.01	–	48.06 ± 0.07	0.345 ± 0.02	0.208 ± 0.01	0.176 ± 0.03	0.287 ± 0.01
	EtOAc	4.24 ± 0.00	–	11.23 ± 0.01	0.283 ± 0.01	0.280 ± 0.00	0.156 ± 0.02	0.634 ± 0.05
	MeOH	37.97 ± 0.03	30.62 ± 0.24	33.43 ± 0.04	0.533 ± 0.02	0.209 ± 0.01	0.324 ± 0.01	0.637 ± 0.02
*Geranium favosum*	DCM	16.38 ± 0.00	–	30.99 ± 0.03	0.453 ± 0.03	0.390 ± 0.02	0.254 ± 0.02	0.210 ± 0.12
	EtOAc	12.17 ± 0.01	–	13.13 ± 0.08	1.029 ± 0.01	0.613 ± 0.12	0.223 ± 0.12	0.389 ± 0.01
	MeOH	92.06 ± 0.00	55.73 ± 0.16	4.25 ± 0.08	2.088 ± 0.08	0.176 ± 0.01	1.738 ± 0.05	0.479 ± 0.00
*Kalanchoe glaucescens*	DCM	11.38 ± 0.00	–	30.99 ± 0.03	0.433 ± 0.03	0.330 ± 0.02	0.153 ± 0.01	0.364 ± 0.08
	EtOAc	12.17 ± 0.01	–	13.13 ± 0.08	1.029 ± 0.01	0.716 ± 0.12	0.301 ± 0.01	1.351 ± 0.02
	MeOH	72.95 ± 0.04	56.44 ± 0.15	4.25 ± 0.08	0.088 ± 0.07	0.166 ± 0.05	0.678 ± 0.02	0.260 ± 0.02
*Lavandula stricta*	DCM	9.99 ± 0.04	–	54.46 ± 0.30	0.290 ± 0.02	0.261 ± 0.01	0.252 ± 0.01	0.127 ± 0.01
	EtOAc	17.66 ± 0.02	10.62 ± 0.35	31.89 ± 0.05	0.325 ± 0.01	0.333 ± 0.01	0.173 ± 0.02	0.505 ± 0.01
	MeOH	41.81 ± 0.02	52.73 ± 0.13	–	0.831 ± 0.09	0.481 ± 0.02	0.456 ± 0.08	0.868 ± 0.04
*Leucas nubica*	DCM	3.76 ± 0.30	2.91 ± 0.34	33.16 ± 0.05	0.319 ± 0.00	0.281 ± 0.01	0.216 ± 0.02	0.400 ± 0.12
	EtOAc	14.77 ± 0.13	–	48.47 ± 0.56	0.413 ± 0.01	0.361 ± 0.12	0.282 ± 0.01	0.517 ± 0.01
	MeOH	48.38 ± 0.05	47.18 ± 0.18	34.03 ± 0.07	1.177 ± 0.09	0.118 ± 0.00	1.015 ± 0.05	0.580 ± 0.01
*Malva parviflora*	DCM	9.66 ± 0.01	–	–	0.423 ± 0.00	0.171 ± 0.00	0.211 ± 0.01	0.558 ± 0.02
	EtOAc	5.89 ± 0.03	–	–	0.313 ± 0.06	0.123 ± 0.00	0.244 ± 0.01	0.952 ± 0.02
	MeOH	13.94 ± 0.06	61.62 ± 0.25	22.51 ± 0.13	0.362 ± 0.07	0.161 ± 0.00	0.337 ± 0.02	0.557 ± 0.04
*Oxalis anthelmintica*	DCM	34.94 ± 0.04	10.79 ± 0.31	16.09 ± 0.09	0.361 ± 0.01	0.249 ± 0.01	0.267 ± 0.47	0.966 ± 0.02
	EtOAc	36.90 ± 0.02	21.99 ± 0.26	14.85 ± 0.08	0.352 ± 0.03	0.324 ± 0.01	0.191 ± 0.02	0.625 ± 0.01
	MeOH	30.83 ± 0.03	39.22 ± 0.22	23.82 ± 0.05	0.474 ± 0.03	0.317 ± 0.01	0.365 ± 0.12	0.460 ± 0.03
*Rumex vesicarius*	DCM	8.41 ± 0.04	–	12.47 ± 0.06	0.405 ± 0.02	0.211 ± 0.00	0.203 ± 0.02	0.819 ± 0.02
	EtOAc	11.76 ± 0.02	–	–	0.407 ± 0.01	0.309 ± 0.00	0.279 ± 0.02	0.808 ± 0.08
	MeOH	46.78 ± 0.25	47.90 ± 0.18	2.49 ± 0.01	0.899 ± 0.04	0.154 ± 0.02	0.845 ± 0.01	0.507 ± 0.04
*Scrophularia arguta*	DCM	5.85 ± 0.04	–	5.31 ± 0.03	0.360 ± 0.01	0.010 ± 0.001	0.203 ± 0.01	0.467 ± 0.02
	EtOAc	9.42 ± 0.03	6.78 ± 0.32	21.30 ± 0.03	0.359 ± 0.01	0.335 ± 0.06	0.249 ± 0.01	1.824 ± 0.01
	MeOH	34.86 ± 0.07	40.07 ± 0.21	26.85 ± 0.06	0.739 ± 0.05	0.363 ± 0.01	0.724 ± 0.02	0.729 ± 0.12
*Senna alexandrina*	DCM	10.64 ± 0.01	–	–	0.335 ± 0.01	0.091 ± 0.01	0.217 ± 0.30	0.623 ± 0.04
	EtOAc	12.05 ± 0.03	–	–	0.362 ± 0.00	0.079 ± 0.01	0.175 ± 0.02	0.887 ± 0.02
	MeOH	18.37 ± 0.05	49.45 ± 0.19	6.10 ± 0.09	0.439 ± 0.01	0.127 ± 0.03	0.331 ± 0.01	0.373 ± 0.01
*Trianthema portulacastrum*	DCM	21.01 ± 0.14	–	–	0.385 ± 0.04	0.431 ± 2.67	0.239 ± 0.01	1.036 ± 0.02
	EtOAc	12.44 ± 0.02	–	–	0.384 ± 0.01	0.373 ± 0.01	0.253 ± 0.02	1.052 ± 0.12
	MeOH	47.61 ± 0.02	29.77 ± 0.24	26.85 ± 0.03	0.755 ± 0.04	0.428 ± 0.00	0.597 ± 0.01	0.441 ± 0.01
*Umbilicus botryoides*	DCM	25.42 ± 0.33	0.76 ± 0.33	33.90 ± 0.01	0.415 ± 0.06	0.431 ± 2.67	0.117 ± 0.02	11.123 ± 0.94
	EtOAc	13.61 ± 0.04	–	36.44 ± 0.03	0.410 ± 0.01	0.373 ± 0.01	0.262 ± 0.12	1.023 ± 0.02
	MeOH	24.15 ± 0.04	52.43 ± 0.18	2.59 ± 0.10	0.353 ± 0.01	0.428 ± 0.01	0.244 ± 0.01	0.154 ± 0.02
References^f^		90.13 ± 0.31	68.32 ± 0.99	61.87 ± 0.98	1.491 ± 0.041	0.782 ± 0.13		

^a^Standard deviation (n = 3)

^b^Absorbance values are given. Higher absorbance indicates greater antioxidant activity

^c^Data expressed in mg equivalent of gallic acid (GAE) to 1 g of extract

^d^Data expressed in mg equivalent of quercetin to 1 g of extract

^e^No activity

^f^References are quercetin for DPPH and DMPD scavenging effect, EDTA for metal-chelating capacity, chlorogenic acid for FRAP, and quercetin for PRAP

The DMPD radical scavenging effect of the extracts varied from none to 61.62 ± 0.25% and the reference (quercetin) gave 68.32 ± 0.99%. The highest scavenging activity against DMPD was observed for the MeOH extract of *M. parviflora* (61.62 ± 0.25%) followed by *K. glaucescens* (56.44 ± 0.15%) and *G. favosum* (55.73 ± 0.15%), respectively.

Results of the metal-chelation capacity of the extracts varied from none to 68.33 ± 0.43%, where the MeOH fruit extract of *A. canariense* demonstrated the highest activity. The EtOAc extracts of *C. barbatus* (67.23 ± 0.06%) and *A. ciliaris* (62.99 ± 0.04%) displayed a good activity comparable to that of the reference (61.87 ± 0.98%).

The FRAP absorbance values of the extracts varied from 0.236 ± 0.007 to 2.088 ± 0.08. Only the MeOH extract of *G. favosum* (2.088 ± 0.08) revealed a strong FRAP activity comparable to that of the reference. The EtOAc (1.029 ± 0.01) and MeOH extracts of *L. nubica* (1.177 ± 0.09) also showed good activities.

The PRAP values of the extracts varied from 0.010 ± 0.00 to 0.716 ± 0.12. The EtOAc extract of *K. glaucescens* (0.716 ± 0.12) gave an interesting activity comparable to that observed by the reference (0.782 ± 0.13) followed by the DMC and EtOAc extracts of *A. ciliaris* (0.649 ± 0.03) and *G. favosum* (0.613 ± 0.12), respectively.

In summary, the highest DPPH scavenging activity and FRAP capacity was obtained from *G. favosum* MeOH extract. The best metal chelation was observed for the MeOH extract of *A. canariense* fruits and EtOAc extract of *C. barbatus*. The best result for the DMPD assay was obtained from the MeOH extract of *M. parviflora*. The EtOAc extract of *K. glaucescens* demonstrated the highest PRAP activity. Furthermore, most of these plants contain considerable amount of polyphenols and flavonoids suggesting their contribution in their antioxidant properties. On the other hand, the DCM extract of *U. botryoides* contained the highest total flavonoid content, whereas it displayed a weak antioxidant activity suggesting that the flavonoids present might possess other activities rather than being antioxidant.

**Fig. 1:**
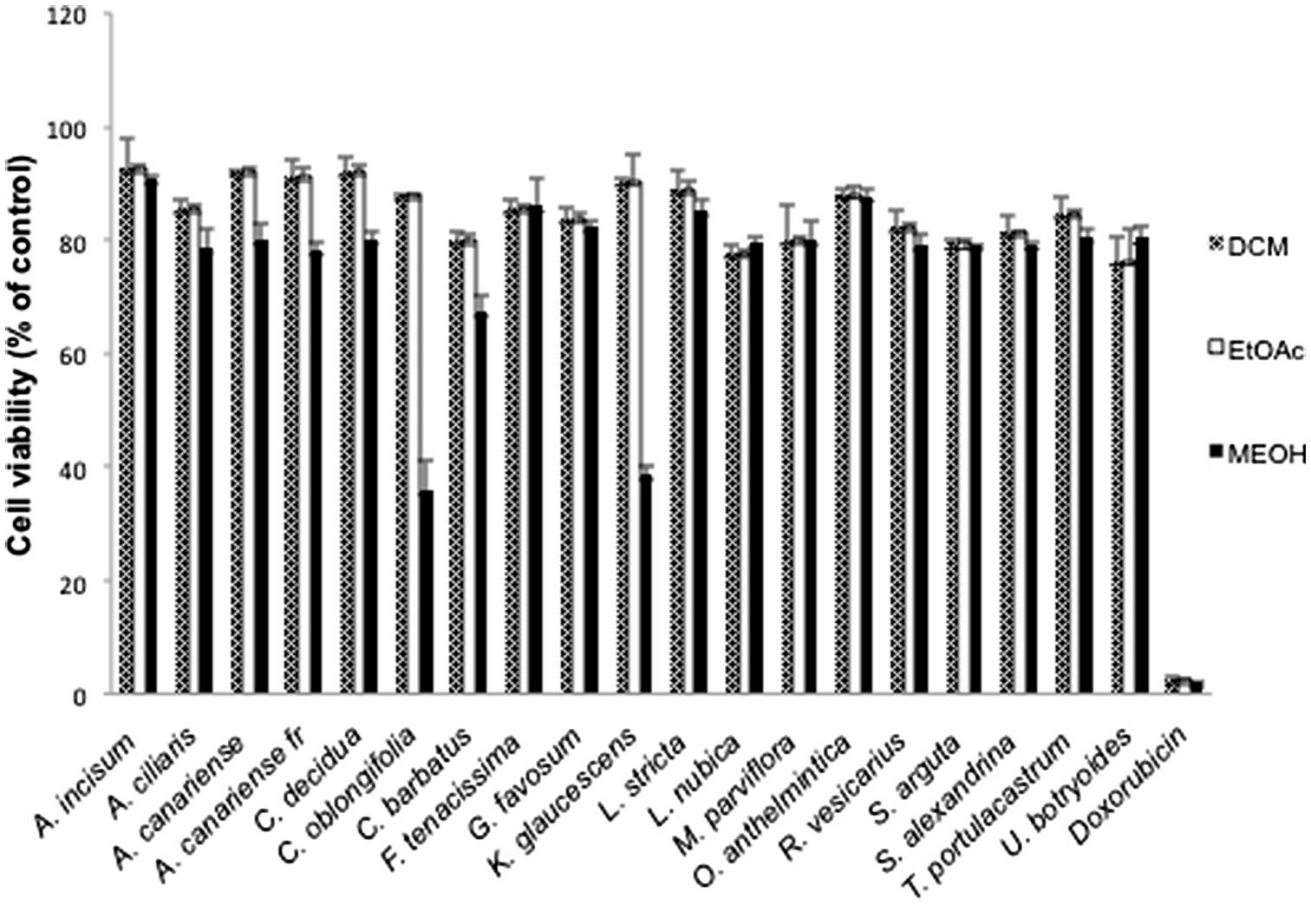
Cytotoxicity of 57 crude extracts from plants selected from a Sudanese region towards CCRF-CEM tumor cells at a fixed concentration of 10 mg/mL as determined by the resazurin assay. Doxorubicin, which was used as a control drug, exhibited a viability of 6% towards the CCRF-CEM-cells at concentration of 10 mg/mL

A preliminary screening of the 57 crude extracts (at concentration of 10 mg/mL) from 18 plant species towards human CCRF-CEM leukemia cells was carried out (Fig. 1). Leukemia cells were chosen in this study for initial screenings, because leukemia cells are frequently more sensitive to cytotoxic agents than most other tumor types. Two MeOH extracts showed considerable cytotoxic activity, i.e., *C. oblongifolia* and *K. glaucescens* (< 40% cell viability compared to untreated control). Another MeOH extract (*C. barbatus*) showed weak cytotoxicity towards CCRF-CEM cells (< 70% cell viability). DCM and EtOAc extracts were not cytotoxic (> 80% viability compared to untreated control).

## Discussion

In this study, we investigated randomly selected Sudanese plants from the Erkowit region to estimate their antioxidant activity using several assays as well as their cytotoxicity against CCRF-CEM leukemia cells. Plants remain one of the main sources of natural products for novel drugs. Therefore, it is a straightforward approach to screen plants for their bioactivity.

The antioxidant activity certainly belong to the most frequently investigated bioactivity in the field of pharmacognosy and natural product research. Plants in general need efficient antioxidant mechanisms to survive in an environment, where they are constantly exposed to sunlight. It can be assumed that the antioxidant mechanisms in plants may also exert beneficial effects for human health [17].

Although antioxidant activity per se cannot be considered as pharmacological activity for disease treatment, it nevertheless may be used as clue that plants with strong antioxidant activity may also be valuable to treat present investigation, we therefore, first tested the antioxidant activities of our panel of plants from the Erkowit region, and then tested the cytotoxic activity towards human cancer cells.

Indeed, we measured considerable antioxidant activities in this panel of plant extracts. Despite the fact that the methods used have different reaction mechanisms and do not necessarily measure the same activities [18], they clearly indicated that the studied plants possess variable antioxidant activity. The antioxidant activity of many, but not all plants is novel and described here for the first time. No previous study on the chemical constituents and biological activity of *G. favosum* has been reported so far, whereas, among other *Geranium* species, the whole plant of *G. wallichianum* possessed a strong antioxidant activity, from which ursolic acid, β-sitosterol, stigmasterol, β-sitosterol galactoside, herniarin, and 2,4,6-trihydroxyethylbenzoate were isolated [19]. Furthermore, our result of antioxidant activity of *M. parviflora* supported that obtained by Afolayan et al. [20].

The antioxidant activity of the leaves of *A. canariense* was previously evaluated by Al-Laith et al. [21], and the presence of alkaloids, coumarins, saponins, tannins, flavonoids, steroids, and triterpenes was also reported [22, 23]. No information on the antioxidant activity of *C. barbatus* and *A. ciliaris* was reported as yet. However, most phytochemical studies were carried out with roots of this plant, where diterpenoid forskolin and its derivatives were identified [24, 25]. Only the antibacterial, cytotoxicity and larvicidal properties of the latter were evaluated thus far [26, 27]. Although many plants belonging to the genus *Kalanchoe* possess various biological activities and are rich in secondary metabolites [28], no information on the biological activity and constituents of *K. glaucescens* is available up to date.

The prescreening of plant extracts may represent the first step in the drug development process. Since several decades, the National Cancer Institute (USA) has been screening innumerous extracts and phytochemicals. Murine leukemia cell line P-388 have been used, before an enlarged screening panel to tumors cell lines of other origins has been applied for cytotoxicity testing [29]. The cytotoxicity screening of the present study revealed that only two extracts were cytotoxic, as they inhibited the proliferation of human CCRF-CEM leukemia cells by more than 50% following incubation for 72 h [30–32]. These extracts were the MeOH extracts of *C. oblongifolia* and *K. glaucescens* suggesting that the active components might be possibly polar. A previous study revealed that a furoclerodane derivative, e.g., croblongifolin, isolated from the stem bark of *Croton oblongifolius* (syn. *Chrozophora oblongifolia*) possessed significant cytotoxicity against various human tumor cell lines including HEP-G2, SW620, CHAGO, KATO3 and BT474 [33].

However, the cytotoxicity of *K. glaucescens* have not been reported as yet. However, other species belonging to the *Kalanchoe* genus exerted remarkable cytotoxicity. For example, bufadienolides isolated from the aerial parts of *K. gracilis* [34] as well as the leaves of *K. pinnata* and *K. duigremontiana* × *tubpaorawas* [35] revealed potential cytotoxicity against several human tumor cell lines. Moreover, flavonoids isolated from leaves of *K. tomentosa* showed cytotoxic activity against P-388 murine leukemia cells [36].

The results of our study showed that antioxidant activity is not completely congruent with cytotoxic activity, but vice versa cytotoxic extracts also revealed antioxidant activity. Therefore, our assumption that the testing of antioxidant activity may serve as preliminary screening, which should be followed by more disease-related assays, such as cytotoxicity testing to identify candidates with anticancer activity may be correct. This hypothesis is also confirmed by numerous reports in the literature showing that cytotoxic herbal extracts and isolated phytochemicals frequently also reveal antioxidant activity [37–42].

To the best of our knowledge, this is the largest study conducted on the antioxidant activity and cytotoxicity of plants from eastern Sudan, in which 18 plant species (57 extracts) were investigated. The results also showed that four of the methods (DPPH, DMPD, FRAP, and PRAP) uniformly identified *G. favosum* as plant with the most antioxidant activity among the studied plants. *K. glaucescens, M. parviflora, A. canariense* and *C. barbatus* also showed an interesting antioxidant potential. *C. oblongifolia* and *K. glaucescens* exerted remarkable cytotoxicity against CCRF-CEM leukemia cells. Moreover, among the 18 plants, 5 (50%) out of 10 plants with medicinal uses were active whereas, 3 (38%) out of 8 plants without known medicinal uses possessed remarkable activities supporting the efficiency of random screenings of plants besides ethno-directed approaches. On the basis of these findings, these plant species should be subjected to further phytochemical analyses to isolate the active compounds and to investigate in depth their modes of action.

## Experimental Section

### Plants Materials

Eighteen plant species were collected from the Erkowit region in eastern Sudan in February/2014. The plant materials were identified and authenticated. Voucher specimens representing each plant were deposited in the herbarium of Botany Department, Faculty of Science, University of Khartoum, Khartoum, Sudan.

### Extract Preparation

100 g of plant samples (whole plant) were dried, ground, and then subjected to sequential cold maceration in stoppered flasks with different organic solvents (hexane, DMC, EtOAc, and MeOH) by gentle shaking overnight at room temperature. The solvents extracted were filtered through Whatman no. 1 filter paper and were evaporated under reduced pressure using rotatory evaporator in order to give crude extracts.

### Antioxidant Activity

#### DPPH Radical Scavenging Activity

The stable 2,2-diphenyl-1-picrylhydrazyl (DPPH) radical scavenging activity was determined by the method of Blois [43]. The samples (30 μL) and reference dissolved in ethanol (75%) were mixed with 2700 μL of DPPH solution (1.5 × 10^−4^ M). Remaining DPPH amount was measured at 520 nm using a Unico 4802 UV–visible double beam spectrophotometer (Dayton, NJ, USA). Quercetin (Sigma, St. Louis, MO, USA) was employed as the reference. Inhibition of DPPH in percent (I%) was calculated as given below:

I% = [(A_blank_ − A_sample_)/A_blank_] × 100, where A_blank_ is the absorbance of the control reaction (containing all reagents except the test sample), and A_sample_ is the absorbance of the extracts/reference.

#### DMPD Radical Scavenging Activity

The assay is based on reduction of the purple-colored radical DMPD^+^ (N,N-dimethyl-*p*-phenylendiamine). According to the method [44], a reagent comprising of 100 mM DMPD, 0.1 M acetate buffer (pH 5.25), and 0.05 M ferric chloride solution, which led to formation of DMPD radical, was freshly prepared and the reagent was equilibrated to an absorbance of 0.900 ± 0.100 at 505 nm. Then, the reagent was mixed up with 50 μL of the extract dilutions and absorbance was taken at 505 nm. Quercetin was employed as the reference. The results were calculated according to the same formula given for DPPH radical scavenging test.

#### Metal-Chelating Capacity

The metal-chelating capacity of the samples through ferrous ion was estimated by the method of Chua et al. [45]. Briefly, dilutions of the extracts were incubated with 2 mM FeCl_2_ solution. The reaction was initiated by the addition of 5 mM ferrozine into the mixture and left standing at ambient temperature for 10 min. The absorbance of the reaction mixture was measured at 562 nm. The ratio of inhibition of ferrozine-Fe^2+^ complex formation was calculated as follows:

I % = [(A_blank_ − A_sample_)/A_blank_] × 100, where A_blank_ is the absorbance of the control reaction (containing only FeCl_2_ and ferrozine), and A_sample_ is the absorbance of the extracts/reference. The reference was employed as ethylenediamine tetraacetic acid (EDTA) in this assay.

#### Ferric-Reducing Antioxidant Power Assay (FRAP)

FRAP of the samples was tested using the assay of Oyaizu [46]. Each sample was mixed with 2500 μL of phosphate buffer (pH 6.6) and 2500 μL of potassium ferricyanide. Later, the mixture was incubated at 50 °C for 20 min and, then, trichloroacetic acid (10%) was added. After the mixture was shaken vigorously, this solution was mixed with distilled water and ferric chloride (0.1%). After 30 min of incubation, absorbance was read at 700 nm. Increased absorbance of the reaction meant increased reducing power and compared to that of chlorogenic acid (Sigma, St. Louis, MO, USA) as the reference.

#### Phosphomolibdenum-Reducing Antioxidant Power (PRAP) Assay

In order to perform PRAP assays, each sample was mixed 10% phosphomolybdic acid solution prepared in ethanol (w/v) [47]. The solution was subsequently subjected to incubation at 80 °C for 30 min and the absorbance was read at 600 nm. Increased absorbance of the reaction meant increased reducing power and compared to that of quercetin as the reference.

### Cytotoxicity

#### Cell Line

Human CCRF-CEM leukemia cell were maintained in RPMI 1640 (Life Technologies) supplemented with 10% FCS in humidified 5% CO_2_ atmosphere at 37 °C. All experiments were done with cells in the logarithmic growth phase.

#### Resazurin Growth Inhibition Assay

The in vitro response to drugs was evaluated by means of growth inhibition resazurin reduction assay to assess the cytotoxicity of the test samples towards the human drug sensitive cancer cell lines (CCRF-CEM) [48, 49]. The assay is based on reduction of the indicator dye, resazurin, to the highly fluorescent resorufin by viable cells. Non-viable cells rapidly lose the metabolic capacity to reduce resazurin and thus produce no fluorescent signal. Briefly, the extracts were dissolved in dimethyl sulfoxide (DMSO) and diluted with RPMI medium to give an initial concentration of 20 mg/mL of various extracts. The cells were plated at a density of 1 × 10^4^ cells/well in a 96-well plate in a total volume of 100 μL. The extracts at a concentration of 20 mg/mL were then added immediately in an additional 100 μL of culture medium to obtain a total volume of 200 μL, therefore, reducing the initial concentration of each extract by half to only 10 mg/mL. After 72 h incubation at 37 °C, 5% CO_2_ and 95% relative humidity, 20 mL resazurin (Sigma-Aldrich, Schnelldorf, Germany) 0.01% w/v in double-distilled water (dd-H_2_O) was added to each well and the plates incubated for a further 4 h. Fluorescence was measured on an Infinite M2000 Pro™ plate reader (Tecan, Crailsheim, Germany) using an excitation wavelength of 544 nm and an emission wavelength of 590 nm. Each assay was done at least twice, with 6 replicate each. The viability was evaluated based on a comparison with untreated cells.

#### Determination of Total Phenol Content

Phenolic content of the extracts was determined in accordance with Folin–Ciocalteau’s method [50]. In brief, a number of dilutions of gallic acid dissolved in ethanol (75%) were obtained to prepare a calibration curve. The extracts and gallic acid dilutions were mixed with 750 μL of Folin–Ciocalteau’s reagent and 600 μL of sodium carbonate in test tubes. The tubes were then vortexed and incubated at 40 °C for 30 min. Afterwards, the absorption was measured at 760 nm. The total phenol content of the extracts was expressed as gallic acid equivalents (mg/g extract).

#### Determination of Total Flavonoid Content

Total flavonoid content of the extracts was calculated by aluminium chloride colorimetric method [51]. To sum up, a number of dilutions of quercetin dissolved in ethanol (75%) were obtained to prepare a calibration curve. Then, the extracts and quercetin dilutions were mixed with 95% ethanol, aluminium chloride reagent, 100 μL of sodium acetate as well as distilled water. Following incubation for 30 min at room temperature, absorbance of the reaction mixtures was measured at wavelength of 415 nm. The flavonoid content of the extracts was expressed as quercetin equivalents (mg/g extract).

#### Statistical Analysis

All data were expressed as the mean standard error (± SEM) of triplicate analysis.
